# Social exclusion increases the executive function of attention networks

**DOI:** 10.1038/s41598-021-86385-x

**Published:** 2021-05-04

**Authors:** Huoyin Zhang, Shiyunmeng Zhang, Jiachen Lu, Yi Lei, Hong Li

**Affiliations:** 1 Institute for Brain and Psychological Sciences, Sichuan Normal University, No. 5, Jing’an Road, Jinjiang District, Chengdu, 610068 China; 2 College of Psychology and Society, University of Shenzhen, Shenzhen, 518067 China; 3 School of Psychology, South China Normal University, Guangzhou, 510631 China

**Keywords:** Psychology, Human behaviour

## Abstract

Previous studies in humans have shown that brain regions activating social exclusion overlap with those related to attention. However, in the context of social exclusion, how does behavioral monitoring affect individual behavior? In this study, we used the Cyberball game to induce the social exclusion effect in a group of participants. To explore the influence of social exclusion on the attention network, we administered the Attention Network Test (ANT) and compared results for the three subsystems of the attention network (orienting, alerting, and executive control) between exclusion (N = 60) and inclusion (N = 60) groups. Compared with the inclusion group, the exclusion group showed shorter overall response time and better executive control performance, but no significant differences in orienting or alerting. The excluded individuals showed a stronger ability to detect and control conflicts. It appears that social exclusion does not always exert a negative influence on individuals. In future research, attention to network can be used as indicators of social exclusion. This may further reveal how social exclusion affects individuals' psychosomatic mechanisms.

## Introduction

Exclusion and rejection are common frustrations in social life. Social exclusion is the process of refusal by others to join a social group, which impedes the need for self-belonging and the desire to be understood in the excluded individual^1^. Social exclusion can profoundly change an individual's physiology, cognition, behavior, and motivation^2^. Research has shown that it often occurs in everyday social interactions and has a strong negative impact on health^3^.

The influence of social exclusion on brain function is mainly concentrated in the right ventrolateral prefrontal cortex (RVLPFC) and anterior cingulate cortex (ACC)^4^. Chester and De Wall (2014) found that interfering with the ACC affects the outcome of social exclusion; by inhibiting ACC activity, the RVLPFC can reduce the pain caused by social exclusion. Thus, RVLPFC activity is negatively correlated with self-reported social pain^4^. Producing social pain has also been shown to change ACC^5^. Imaging data has indicated that RVLPFC activation significantly increases after individuals experience social exclusion.

The ACC has been shown to play a key role in the executive control system for attention. Previous studies have suggested that as social exclusion occupies the brain resources needed for self-regulation, resources used in behavior monitoring are reduced. Thus, social exclusion may reduce the efficiency of individual executive control^6^. This was supported by a study showing that when the Future Rejection paradigm was used, social rejection leads to lower levels of cognitive control^7^. However, an event-related potential (ERP) study found that social exclusion activates brain regions that underlie cognitive control. Thus, it stimulates a certain degree of emotional regulation, which significantly improves cognitive control^8^. The results of these two studies appear to be in direct conflict, so it is not yet clear how brain regions related to both behavioral monitoring and social exclusion can impact individual actions. Further, systems such as executive control are subordinate to the attention network, thus obscuring the precise effects of social exclusion in terms of broader attentional function.

We used the following paradigm to explore the relationship between social exclusion and the attention network. We refer to an attention network that has been defined by predecessors as having three subsystems: alerting, orienting, and executive attention^9^. Alerting is the most basic aspect of the attention network. When active, the alerting attention system maintains a high level of arousal and makes the body sufficiently prepared for detecting events in the environment. The orientating attention system guides an individual to choose one of the many incoming stimuli as an object for processing. The executive attention system is the dominant subsystem which is mainly responsible for controlling attention by switching between multiple cognitive tasks and conflict management^10^.

In a visual search task experiment in a previous study, social exclusion showed no effect on the behavioral results of selective attention, but there were differences in the EEG results. Francesco Bossi et al. (2018) found that selective attention responses were increased in behavioral outcomes in the context of social exclusion caused by the Cyberball paradigm^11^. Wang Chaolun et al. (2019) found that social information has different effects on the attention network model than non-social information, with significant effects on vigilance, orientation, and executive control sub-networks^12^.

Based on previous studies, we speculate that social exclusion may promote all subsystems of attention network rather than only select subsystems^13^. Interestingly, there was no significant difference in Xu's behavioral results in terms of the unconscious control of attention by social exclusion; the reaction time of their exclusion group was shorter. Unconscious control includes unconscious inhibitory control and unconscious conflict adaptation. It is a process of control distinct from conscious attention through effort. Previous researchers have mostly focused on the effects of social exclusion on selective attention and cognitive control while few have studied the relationship between social exclusion and the three systems of the attention network as a whole.

Fan (2002) used the Attention Network Task (ANT) paradigm to simultaneously distinguish and explore the processing of each of the three subsystems^9^. This paradigm includes the Flanker Task and the Spatial Cue task, which have been widely used in pathology studies on the attention network^14^. In this study, we focused on the performance of individuals on the ANT after social exclusion and determined the effect of social rejection on the whole attention network as well as each individual subsystem. We also sought to determine whether social exclusion has a beneficial or deleterious effect on attention. We assumed that social exclusion has a certain influence on the response time and correctness rate of individuals' ANT performance. The social exclusion group and social inclusion group showed significant differences across the three subsystems, as discussed in detail below.

## Methods

### Participants

One hundred and twenty college students were randomly assigned to the exclusion group (n = 60; 28 males and 32 females; mean age: 21.3 ± 1.12 years) or the inclusion group (n = 60; 30 males and 30 females; mean age: 20.8 ± 1.08 years). All were non-psychology or related majors and right-handed. The experimental procedure was approved by the Ethics Committee at the Institute for Brain and Psychological Sciences of Sichuan Normal University. All procedures performed in studies involving human participants conformed to the Code of Ethics of the World Medical Association (Declaration of Helsinki). All participants provided their written informed consent before the experiment and were paid 30 yuan (about 4 USD) after the experiment.

### Materials

#### Cyberball game

To induce the experience of exclusion, participants played a modified version of the Cyberball paradigm (Cyberball 5.0)^15^. In this computer game, participants toss a ball with two other players who are represented on-screen by animated icons. Although the participants are led to believe that the other players were real people, they are in fact programmed virtual players. We created social exclusion or inclusion scenarios by controlling the percentage of players who catch the ball^8^. The online version of Cyberball 5.0 was run using an HTML5 compatible web browser (Google Chrome). Written instructions were given to participants to inform them that the game “tests imagination” and that they would be playing online against “other students”. The backdrop for the game was customized with a photograph of a school playground. Each iteration of the game included 30 ball tosses between the players.

In the inclusion group, the pseudo player tossed the ball to the participant and the other virtual player with the same probability. In the exclusion group, conversely, the participants only received the ball one time at the beginning of the experiment^15^. The Cyberball game has been developed with online and stand-alone options and is widely used in cognitive neuroscience research on social exclusion.

#### ANT paradigm

The ANT task can effectively measure three attention network subsystems. It is easy to understand and operate, as mentioned above, has been widely used in experiments related to attentional networks. In the ANT paradigm, the participant needs to complete two tasks: The Flanker Task^16,17^ and the Spatial Cue task.

**Figure 1:**
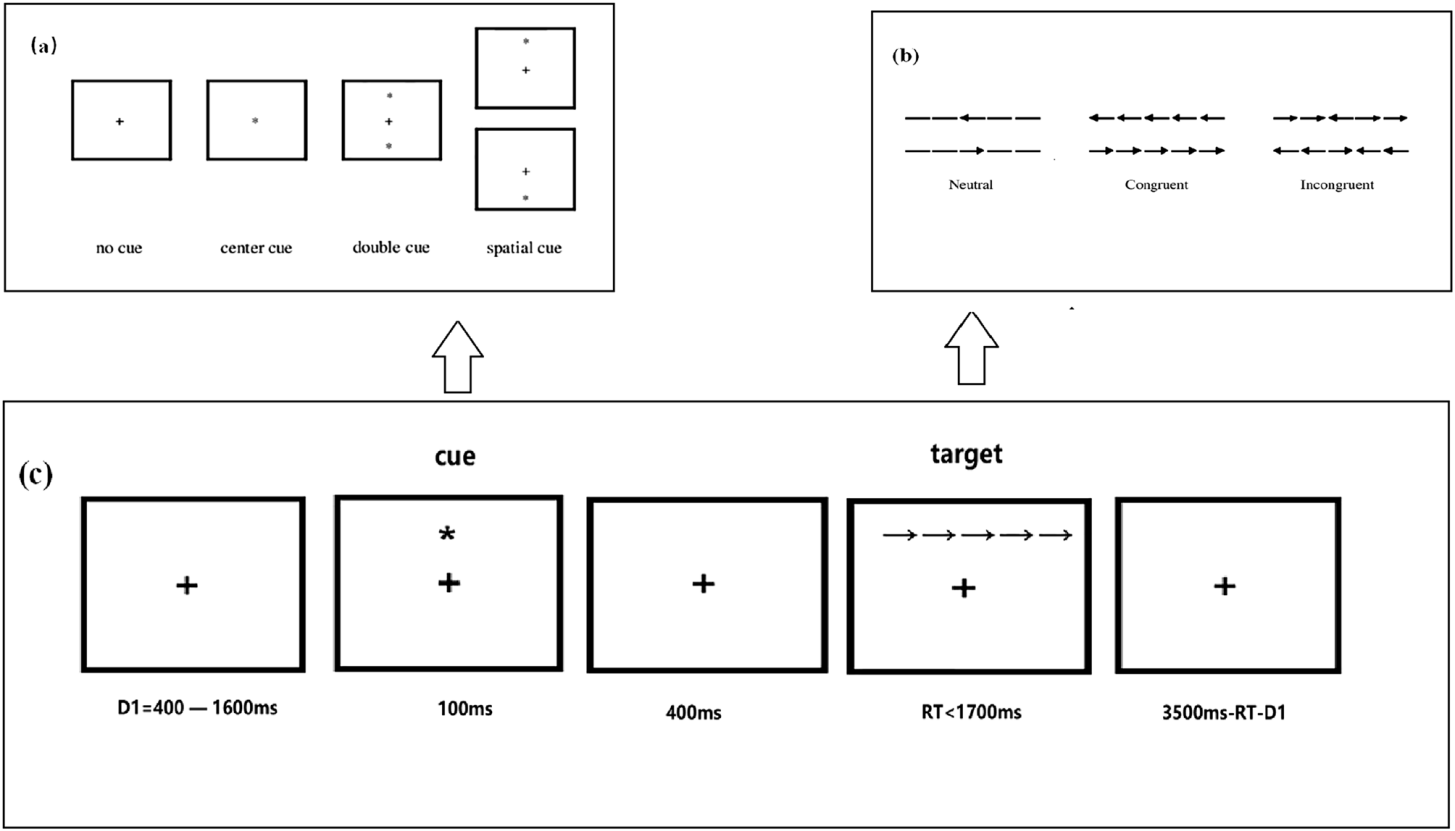
(**a**) No-cue, central cue, double cue, and spatial cue conditions; (**b**) neutral, congruent, and incongruent flanker conditions; (**c**) example trial process composed of fixation point, cue condition, flanker condition, reaction time, and remaining time indicators.

In the Flanker Task, the participant reacts to the middle arrow in a row of arrows facing left or right by deciding which direction the arrow is pointing. Figure 1c shows the sequence of events for a single trial. The participants are instructed to “Please look at the fixation cross in the middle of the screen. After a certain amount of time, a line of arrows appears above or below the fixation. Please quickly and accurately determine the orientation of the middle arrow. If the arrow is facing left, press the left-arrow key on the keyboard, and if the arrow is facing right, press the right-arrow key”. First, a fixation cross of variable duration (400–1600 ms) is presented. A 50-ms cue then appears to indicate the position of the arrow that follows. After 400-ms stimulus onset asynchrony (SOA), a row of arrows going left or right appears while a target and flankers are presented on the same or opposite locations of the orienting cue and remain on the screen for 1700 ms or until a response is given. After the response, the fixation cross is kept on screen for a variable duration dependent on the initial duration of the fixation cross and the RT of the participant. Each trial lasts 3500 ms.

The experiment was conducted according to a 4 (types of cues) × 3 (flanker conditions) factorial design. The task had three conditions, one in which the direction of the middle arrows matches the other arrows (congruent condition), one in which it is the opposite (incongruent condition), and one in which the surrounding items are not arrows (neutral condition) (Fig. 1b). We measured the executive control function of attention by comparing individual responses to the congruent and incongruent flanker conditions^9^. Our research paradigm is consistent with our predecessors. We established the desired conditions through the ANT paradigm and mathematically formulated the three attention systems as described below^9^. Before the start of each trial, cue stimuli (asterisks) appeared on the screen: no-cue, central cue, double cue, and spatial cue^18^ (Fig. 1a).

Trials with no-cue began with a standard fixation spot (a cross) followed by the flanker stimuli presented above or below the fixation. In trials with a central cue, a cue (an asterisk) appeared in the center of the screen, followed by the flanker task presented above or below the fixation. In the double-cue condition, asterisks appeared above and below the fixation spot followed by the flanker stimuli randomly presented above or below the fixation. In the spatial cue condition, the cue appeared above or below the fixation and matched the position of the subsequent flanker stimuli.

The remaining two attentional networks were assessed by examining differences in response time under different conditions. According to Fan (2002), in the no-cue condition, attention is distributed above and below the fixation point; in the double-cue condition, vigilance is aroused and attention is again distributed above and below the fixation^9^. Therefore, the effect size of the alert subsystem can be calculated as the difference between the average response times for the no-cue and double-cue conditions. The central cue encourages attention toward a location, while spatial cues accurately predict spatial information. Therefore, the effect of the orientation network subsystem can be calculated as the difference between the average response times for the central-cue and spatial-cue conditions.

In this study, we used the ANT paradigm to measure the effect sizes of three subcomponents of the attention network. Therefore:

$$Alerting \;effect = {\text{ RT}}_{no \;cue} {-}{\text{ RT}}_{double \;cue}$$

$$Orienting\; effect = {\text{ RT}}_{center\, cue} {-}{\text{ RT}}_{spatial\; cue}$$

$$Conflict\; effect = {\text{ RT}}_{incongruent} {-}{\text{ RT}}_{congruent}$$

### Experimental program

**Figure 2:**
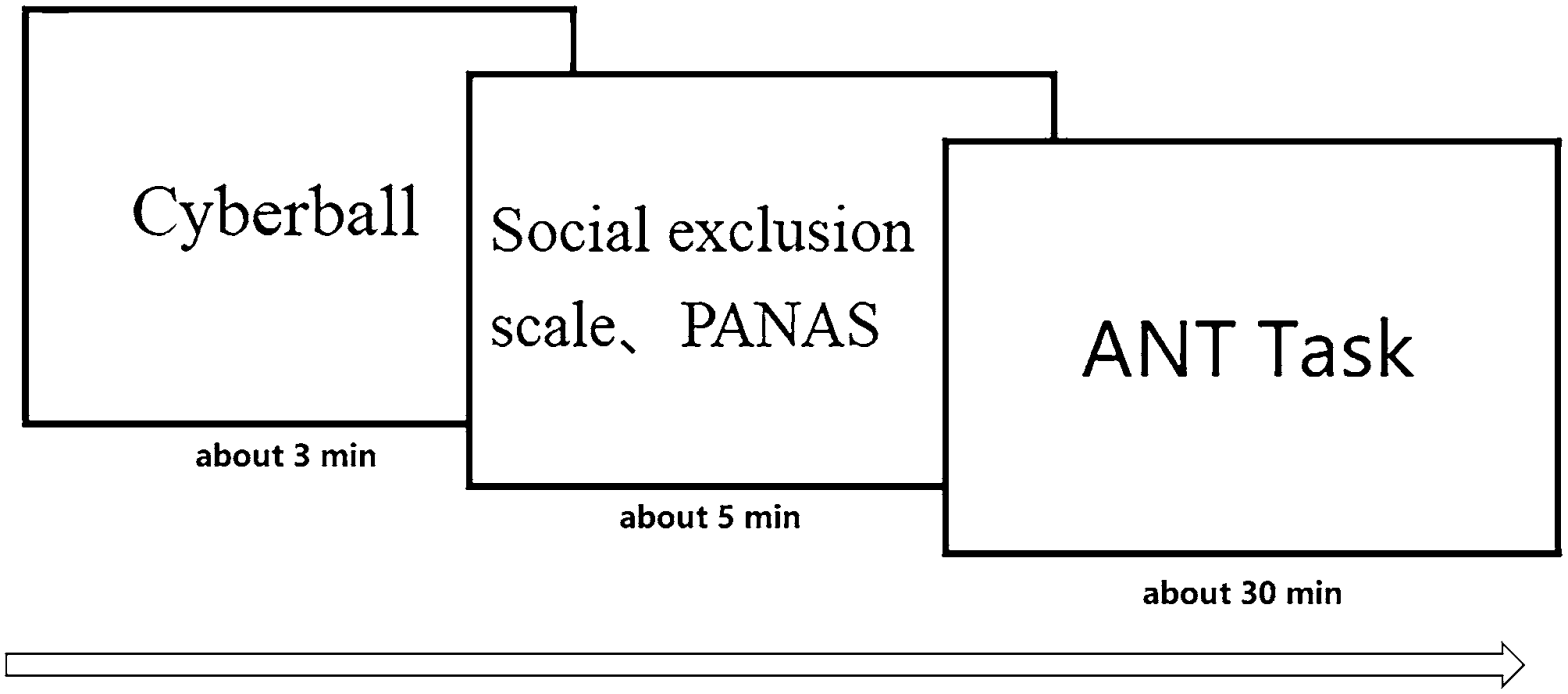
Complete experimental flow.

The steps of this experiment were as follows. (1) Participants were randomly divided into the exclusion and inclusion groups while keeping a relatively balanced sex ratio. (2) Participants played the Cyberball game with two other players. (3) After completing the Cyberball game, the participants filled out the Positive and Negative Affect Schedule (PANAS) and Social Exclusion Scale^19^. (4) Participants performed the ANT including 24 practice exercises to familiarize them, which were not included in the analysis, followed by the formal task. After every 96 trials there was a rest period. The rest duration was determined by the participants themselves (about one minute each). The whole experiment lasted 25–30 min and varied from individual to individual. A flow chart of the complete process is shown in Fig. 2.

### Data analysis

As is general practice, the reaction times (RTs) of error trials and outliers were excluded. Outliers were detected by any data points exceedingly plus or minus three standard deviations. These criteria resulted in a loss of 6.8% of the original data leaving 32,209 remaining observations.

Data analysis was carried out in the R software environment^20^ (Version 3.5.0) for the experiment and questionnaire. We used an independent sample *t* test for the questionnaire. For RT, we first conducted a log-transformation due to its non-normal distribution and then performed a mixed-effect linear regression (LME) on the log-transformed RTs by the *lmer* function of the “lme4” package^21^ (Version 1.1.21) and “lmerTest” package^22^ (Version 3.1.0). The GraphPad Prism8.0.1 was used for visualization (https://www.graphpad.com/). Both the main effects and the interactions of group, cue type, and congruency were entered as fixed effects. Subjects were entered as random intercepts to the model. Similar analyses were also performed on the correctness rate. Significance was evaluated using the Kenward–Rogers approximation for degrees of freedom^23,24^. For all analyses, the alpha was set at *p* < 0.05 (two-tailed). Whenever necessary, *p*-values from multiple comparisons were FDR-corrected^25,26^.

According to Fan et al. (2005), there are three attention networks to be measured in the ANT: Alerting effect (RT no cue – RT center cue), orienting effect (RT center cue – RT spatial cue), and conflict effect (RT incongruent -RT congruent)^16^. Similar linear mixed model analyses were also performed on the three attention networks except where both the main effects and the interaction of the group and attention network were entered as fixed effects.

### Declaration of ethics

All procedures performed involving human participants were in accordance with the ethical standards of the institutional research committee and with the 1964 Helsinki Declaration and its later amendments or comparable ethical standards. The experimental procedure in the article was approved by the Ethics Committee at the Institute for Brain and Psychological Sciences of Sichuan Normal University. In response to the Standard Reviewer Disclosure Request (2014) endorsed by the Center for Open Science (https://osf.io/chx76/), the authors confirm that in this manuscript, they have reported all measures, conditions, data exclusions, and methods for determining sample sizes.

### Informed consent

Informed consent was obtained from all individual adult participants included in the study.

## Results

### Rating scale

We found no significant difference between the exclusion and inclusion groups on the PANAS (*t*_(95)_ = 1.25, *p* = 0.21, *Cohen’s d* = 0.24). We found no significant differences between the groups for positive emotions (*t*_(110)_ = 0.43, *p* = 0.67, *Cohen’s d* = 0.05). In contrast, scores on the exclusion scale were significantly higher in the exclusion group than in the inclusion group (*t*_(107)_ = 7.3, *p* < 0.001, *Cohen’s d* = 1.64).

### RT

We carried out LMEs on the performance of each condition in the ANT with group, cue type, and congruency treated as fixed effects. The subject was treated as a random effect.

The model revealed significant main effects of congruency in RT (*F* (2, 32,067) = 4612.56, *p* < 0.001) and cue type (*F* (3, 32,067) = 1491.95, *p* < 0.001), as well as a significant two-way interaction between cue and congruency (*F* (6, 32,067) = 51.20, *p* < 0.001), between cue and group (*F* (3, 32,067) = 5.08, *p* = 0.002), and between congruency and group (*F* (2, 32,067) = 11.10, *p* < 0.001). In addition, RT in the incongruent condition was longer than that in the congruent condition (*β* = 0.06, *SE* = 0.0007, *z* = 82.81, *p* < 0.001) or neutral condition (*β* = 0.06, *SE* = 0.0007, *z* = 84.43, *p* < 0.001). RT in the neutral condition and in the congruent condition showed no significant difference (*β* = − 0.001, *SE* = 0.0007, *z* = − 1.55, *p* = 0.26). Moreover, RTs in the no cue condition > in the central cue condition > in the double cue condition > in the spatial cue condition (all *β* > 0.01, all *SE* = 0.0008, all *z* > 4.39, all *p* < 0.001).

Further analysis on the interaction between cue type and congruency revealed that under all cue conditions, RTs in the incongruent condition were longer than those in the congruent condition (all *β* > 0.04, all *SE* = 0.001, all *z* > 28.85, all *p* < 0.001) or neutral condition (all *β* > 0.04, all *SE* = 0.001, all *z* > 29.38, all *p* < 0.001). Under all cue conditions, RTs in the neutral condition and those in the congruent condition were not significantly different (all *β* > − 0.0007, all *SE* = 0.001, all *z* > − 1.61, all *p* > 0.24). Only under incongruent conditions were RTs in the inclusion group longer than those in the exclusion group (*β* = 0.01, *SE* = 0.007, *z* = 1.71, *p* = 0.09). RTs in the inclusion group and those in the exclusion group were not significant under neutral conditions (*β* = 0.006, *SE* = 0.007, *z* = 0.85,* p* = 0.39) or congruent conditions (*β* = 0.006, *SE* = 0.007, *z* = 0.83, *p* = 0.40).

Further analysis on the interaction between group and cue type revealed that RTs in the inclusion group were longer than those in the exclusion group only under the spatial cue conditions (*β* = 0.01, *SE* = 0.007, *z* = 1.69, *p* = 0.09). RTs in the inclusion group and those in the exclusion group were not significant under the no-cue condition, congruent condition, or center cue conditions (all *β* > 0.005, all *SE* = 0.007, all *z* > 0.83, all *p* > 0.31).

### Correctness rate

Regarding correctness rate, the model revealed significant main effects of congruency (*F* (2, 1298) = 91.17, *p* < 0.001) as well as significant two-way interactions between group and congruency (*F* (2, 1298) = 12.18, *p* < 0.001). The incongruent condition showed less accurate performance than the congruent (*β* = − 0.04, *SE* = 0.003, *t* = − 11.85, *p* < 0.001) or neutral (*β* = 0.04, *SE* = 0.003, *t* = − 11.53, *p* < 0.001) conditions. Correctness rate values in the neutral condition and those in the congruent condition were not significant (*β* = − 0.001, *SE* = 0.003, *t* = − 31, *p* = 0.95).

Further analysis on the interaction between group and congruency revealed that correctness rate in the inclusion group was more accurate than in the exclusion group. Only under incongruent conditions (*β* = 0.02, *SE* = 0.009, *t* = 2.45, *p* = 0.02. correctness rate values in the inclusion and exclusion groups were not significantly different under neutral conditions (*β* = − 0.007, *SE* = 0.009, *t* = − 0.83, *p* = 0.41) or congruent conditions (*β* = − 0.006, *SE* = 0.009, *t* = − 0.67, *p* = 0.50).

### Group-wise differences in ANT scores

**Figure 3:**
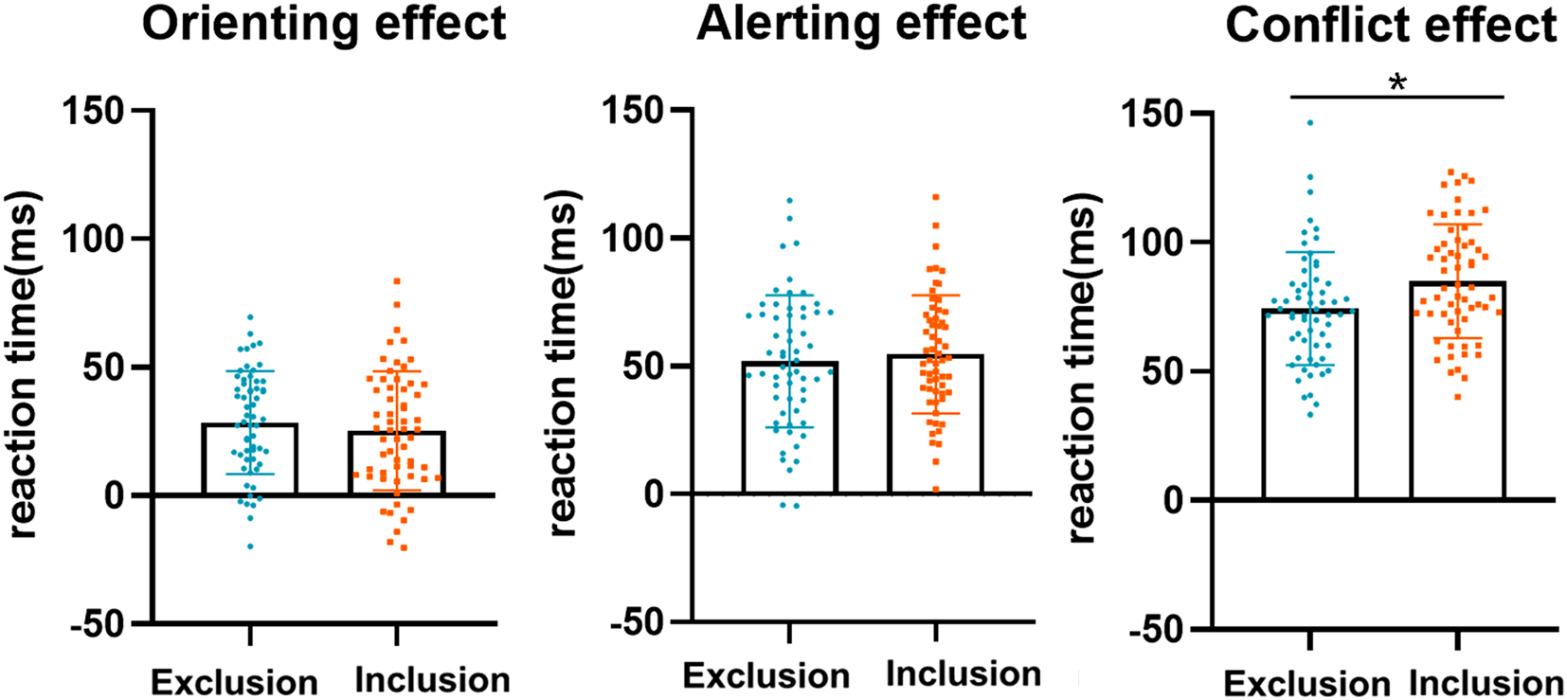
Mean and standard deviation of three effects: exclusion (left) and inclusion (right) groups differ significantly only in conflict effect (*p < 0.05). The result of attentional network scores using the lmer function of the “lme4” package^21^ (Version 1.1.21) and “lmerTest” package^22^ (Version 3.1.0).

To address the group comparisons on attentional network scores, we carried out LMEs on the performance of each condition in the ANT with group and attentional network treated as fixed effects with subject as a random effect. The model revealed a significant interaction between the group and attentional network (*F* (2, 236) = 2.99, *p* = 0.052). As shown in Fig. 3, further analysis revealed that RT in the inclusion group was longer than in the exclusion group only in terms of the conflict effect (*β* = 10.66, *SE* = 4.15, *t* = 2.57, *p* = 0.01); RTs in the inclusion and exclusion groups were not significant in terms of the orienting effect (*β* = − 3.34, *SE* = 4.15, *t* = − 0.80, *p* = 0.42) or alerting effect (*β* = 2.85, *SE* = 4.15, *t* = 0.69, *p* = 0.50).

## Discussion

This study explored whether social exclusion affects the three subsystems (alerting, orienting, and executive control) and the whole of the attention network. The results were partially in line with our expectations. On this model, the social exclusion group shows shorter responses than the social inclusion group.

The main effects of cue type and flanker condition were significant in terms of reaction time measurements, indicating that the ANT paradigm effectively measured the attention network. The interaction between group and flanker condition was also significant, indicating that attentional orientation differed between the groups. The interaction between cue type and flanker condition was significant. In terms of correctness rate, we found no significant difference between the exclusion and inclusion groups, but the interaction between cue type and flanker condition was significant. The interaction between group and direction was significant; the correctness rate of the inclusion group was higher than that of the exclusion group in all three directions.

The overall response time for the social exclusion group was shorter than that of the social inclusion group, which is inconsistent with previous studies^7^. In general, social rejection makes the reaction time longer. The cognitive resource depletion theory is likely responsible for this; some of the resources originally allocated to attention appear to be allocated to the self-regulation of social exclusion. In other studies, this resulted in insufficient resources allocated to attention, so the exclusion group assigned less attention to the current task. In our study, social exclusion was caused by the Cyberball paradigm. We hypothesized that the result might be that social rejection induced by the Cyberball paradigm is not enough to impair cognitive resources, but instead activates brain regions involved in self-regulation, shortening response times. In subsequent studies, this can be discussed in more detail in experiments using functional magnetic resonance imaging to identify activated brain regions.

However, correctness rate did not significantly differ between the inclusion and exclusion groups. In recent years, studies have shown that social exclusion causes pain^27,28^. The neural regions that process the emotional components of pain include the ACC and the anterior insula; these regions exhibit greater activation in response to the experience of social exclusion^29,30^. Individuals who suffer from this type of pain have a greater incentive to reconnect with others to relieve their suffering. Additionally, when the reaction time of the exclusion group to the attention system was shortened, their correctness rate decreased compared to the inclusion group as evidenced by a “trade off” between speed and correctness rate. The lack of difference between the two was the result of the combined effect of the two causes. The exclusion group had strong motivation but a short reaction time; their correctness rate did not differ significantly from that of the inclusion group. The correctness rate of the inclusion group, however, was higher than that of the exclusion group in different directions. Under consistent conditions, the correctness rate of the two groups was not affected. This indicates that the consumption of cognitive resources in the incongruent condition makes the correctness rate of the exclusion group decrease. It may also be that the exclusion group inadvertently reduced correctness rate as they attempted to maintain responsiveness.

Among the three subsystems of the attention network, we did not find any significant differences between the exclusion and inclusion groups in regard to alerting and the orienting systems. In contrast, we found that response times for the executive control system were shorter in the exclusion group than in the inclusion group. This marks a slight departure from previous studies in which social exclusion affected selective attention such that alert-system responses were slower.

In this study, alert-system responses did not differ between groups. There is a reasonable explanation for this. Previous researchers speculated that after people experience exclusion, their sense of security in society decreases and they seek to improve their ability to alert themselves in order to cope with environmental emergencies and to ensure their own safety^31^. The regions of the brain responsible for alertness were activated, which offset the depletion of resources caused by social exclusion. In terms of the alert systems of our participants, there were no differences in response time between exclusion and inclusion groups. In addition, due to the larger standard deviation (SD) in the exclusion group, there was a population difference effect. The effects of exclusion on the attention network were divided across three subsystems, so the difference is not as significant as the total; after dispersion, the effect may only be observable in one system.

We found no significant difference in the orientation subsystem between the groups. As observed in previous studies, the orientation system of the attention network is advantageous in identifying self-information^32^. The lack of difference in orientation response times here may have been due to the activation of brain regions in both groups, rather than only in the exclusion group. It is also possible that exclusion makes individuals more sensitive to information given by others, making the orientation system, which should be slower in response, no different from that of the inclusion group.

Fuhrmann et al. (2019) found that social exclusion affects the working memory of teenage women. Working memory is a part of the executive system^33^. Other studies have also demonstrated that social exclusion can enhance the executive control system^8^. Although many have shown that social exclusion has a detrimental effect on inhibition control^6^, here, we found that executive control of the attention network increases with social exclusion. This partially supports the conclusions of Otten and Jonas (2013)^8^.

The ACC is a brain region activated during social exclusion and during executive control^34^. Therefore, social exclusion actually activated executive control function, resulting in a shortened response time for the executive control subsystem of attention in our exclusion group. Additionally, the ACC plays an integral role in regulating emotions while the VMPFC is often associated with the generation and regulation of negative emotions^35,36^, which can be brought on by social exclusion^4,37^. Thus, the emotion-integrated brain regions become activated and the self-regulating functions of the individual do not overreact after a short-term rejection that does not have a large negative impact on the individual^38^. Previous studies have shown that self-regulation is positively correlated with cognitive control^39^. In other words, the ability to self-regulate facilitates the activation and operation of the cognitive control system and improves the executive control of attention in individuals after social exclusion.

## Limitations and future research

The ANT paradigm cited in a previously published article was modified to suggest that different attention networks are not independent but rather are integrated with each other^17,40^. Callejas (2004) used an alarm signal with a high-frequency tone to accelerate response times in participants^41^; the sound of an alarm can indeed affect human behavior^41^. In this study, we adopted the definition given by Fan (2002)^9^, the default premise of which is that the three attention networks are independent of each other.

In addition, the Cyberball game can only stimulate short-term social exclusion in individuals^42,43^. To increase the external validity of the whole experiment and to fully determine whether long-term social exclusion has an irreversible effect on attention, better paradigms for stimulating social exclusion need to be developed. Alternatively, researchers may directly recruit participants with real, long-term experience of social exclusion or campus exclusion. Previous researchers used real images of social pain as experimental material to find that such pain is associated with the right VLPFC^44^. This may be a workable future direction for our research.

In future studies, participants can be asked to complete a personality questionnaire to analyze whether personal characteristics are also factors that influence the experimental results. Questionnaires that may be helpful in this regard include the State-Trait Anxiety Inventory (STAI) and Hamilton Anxiety Scale (HAMA), which can measure anxiety traits prior to participating in a social exclusion experiment. As for our experiment itself, we believe that if the social exclusion effect had been re-tested after the attention paradigm task, the results would have stronger reliability and validity. Intervention to reduce the negative consequences of social exclusion has become a hot research topic in recent years^45^. Human's physiological cycle and body changes also have an impact on the processing of social information. In the future, attention system characteristics could be considered as a measure of treatment efficacy in socially excluded individuals.

## Conclusion

This study was conducted to explore the effects of social exclusion on three systems of attention. The results showed that individuals who experienced social exclusion had shorter response times to the Flanker task in the ANT paradigm, but no difference in correctness rate compared to individuals who did not experience social exclusion. In addition, reaction times in the executive control system were faster in the exclusion group than in the inclusion group, which indicates that the excluded individuals had a stronger ability to detect and control conflicts. Our results also indicate that exclusion did not always exert a negative influence on individuals, which provides a novel perspective on social exclusion. Future studies on social exclusion can be designed to include characteristics of the attention system as indicators of exclusion.
